# Impact of socioeconomic deprivation on rate and cause of death in severe mental illness

**DOI:** 10.1186/s12888-014-0261-4

**Published:** 2014-09-12

**Authors:** Julie Langan Martin, Gary McLean, John Park, Daniel J Martin, Moira Connolly, Stewart W Mercer, Daniel J Smith

**Affiliations:** Institute of Health and Wellbeing, University of Glasgow, Gartnavel Royal Hospital, 1055 Great Western Road, Glasgow, G12 0XH UK; Lead Research Nurse PsyCIS Team, Stobhill Hospital, 300 Balgrayhill Road, Glasgow, G21 3UR UK; Gartnavel Royal Hospital, 1055 Great Western Road, Glasgow, G12 0XH UK; Institute of Health and Wellbeing, University of Glasgow, General Practice & Primary Care, 1 Horselethill Road, Glasgow, G12 9LX UK

**Keywords:** Severe mental illness (SMI), Schizophrenia, Bipolar disorder, Mortality, Deprivation

## Abstract

**Background:**

Socioeconomic status has important associations with disease-specific mortality in the general population. Although individuals with Severe Mental Illnesses (SMI) experience significant premature mortality, the relationship between socioeconomic status and mortality in this group remains under investigated. We aimed to assess the impact of socioeconomic status on rate and cause of death in individuals with SMI (schizophrenia and bipolar disorder) relative to the local (Glasgow) and wider (Scottish) populations.

**Methods:**

Cause and age of death during 2006-2010 inclusive for individuals with schizophrenia or bipolar disorder registered on the Glasgow Psychosis Clinical Information System (PsyCIS) were obtained by linkage to the Scottish General Register Office (GRO). Rate and cause of death by socioeconomic status, measured by Scottish Index of Multiple Deprivation (SIMD), were compared to the Glasgow and Scottish populations.

**Results:**

Death rates were higher in people with SMI across all socioeconomic quintiles compared to the Glasgow and Scottish populations, and persisted when suicide was excluded. Differences were largest in the most deprived quintile (794.6 per 10,000 population vs. 274.7 and 252.4 for Glasgow and Scotland respectively). Cause of death varied by socioeconomic status. For those living in the most deprived quintile, higher drug-related deaths occurred in those with SMI compared to local Glasgow and wider Scottish population rates (12.3% vs. 5.9%, p = <0.001 and 5.1% p = 0.002 respectively). A lower proportion of deaths due to cancer in those with SMI living in the most deprived quintile were also observed, relative to the local Glasgow and wider Scottish populations (12.3% vs. 25.1% p = 0.013 and 26.3% p = <0.001). The proportion of suicides was significantly higher in those with SMI living in the more affluent quintiles relative to Glasgow and Scotland (54.6% vs. 5.8%, p = <0.001 and 5.5%, p = <0.001).

**Conclusions:**

Excess mortality in those with SMI occurred across all socioeconomic quintiles compared to the Glasgow and Scottish populations but was most marked in the most deprived quintiles when suicide was excluded as a cause of death. Further work assessing the impact of socioeconomic status on specific causes of premature mortality in SMI is needed.

## Background

It is recognised that socioeconomic status has a significant impact on health and wellbeing and, with only a few exceptions, health outcomes are generally worse in deprived communities [1]. In Western societies, although there is evidence of equity in access to primary care services across different socioeconomic profiles, there is also evidence of inequalities in access to more specialist services [2,3]. In Scotland, which has had the highest mortality in Western Europe among working age (15-74 years) men and women since the late 1970s [4], premature mortality and inequalities in healthcare remain important [5,6]. In 2010 it was estimated that Scottish male mortality rates were around 20% higher and female working age mortality 30% higher than the Western European mean. Although Scotland has been dubbed the “Sick Man of Europe”, its biggest city Glasgow has even higher mortality rates, along with higher rates of deaths from suicide, drugs and alcohol and lower life expectancy than the rest of Scotland [7]. While it is known that the impact of deprivation on health and mortality is large, the differences in life expectancy in Scotland compared to the rest of Europe (and in Glasgow compared to the rest of Scotland) cannot be fully explained by differences in its deprivation profile alone. These differences which are unexplained by deprivation have been termed the “Scottish” and “Glasgow” Effects.

For individuals with SMI, the increase in standardised mortality rates (SMRs) compared to the general population is substantial [8-11]. On average, men with schizophrenia die 20 years earlier and women die 15 years earlier than the general population [12,13]. For bipolar disorder, life expectancy is estimated to be reduced by approximately 10 years in men and 11 years in women [11]. Furthermore, there is recent evidence from the UK [12], US [13] and Western Australia [14] to suggest that this mortality gap may be getting worse. Although it is recognised that individuals with SMI have more physical health problems than the general population [15,16], there is also evidence of inequalities for both access to and the quality of a range of physical healthcare services [17–21]. In combination with the inequality in access to treatment, it is recognised that SMI individuals tend to experience a greater downward trend in socioeconomic status over time compared to the general population [22]. Despite the important and complex interaction between socioeconomic status and mortality, little work has specifically focused on this relationship for individuals with SMI. Here we describe and compare rates and causes of death for individuals with schizophrenia and bipolar disorder who were registered on the Glasgow Psychosis Clinical Information System (PsyCIS) according to socioeconomic quintile, relative to the local Glasgow population and the wider Scottish population. We seek to determine if as is seen in the general population, increasing deprivation is associated with increased mortality rates and we also examined whether or not the “Glasgow” Effect was present in our cohort of individuals with SMI.

## General methods

### Construction and content of the database

The Glasgow Psychosis Clinical Information System (PsyCIS) is a database of all individuals with psychotic illness who have been in contact with secondary care psychiatric services in Glasgow, Scotland, covering a population of approximately 1 million people. Data were initially collected retrospectively by two research nurses. From August 2005 onwards new cases have been added prospectively to the database using existing patient information management systems. All patients have detailed socio-demographic information including International Statistical Classification of Diseases and Related Health Problems (ICD-10) diagnosis, unique Community Health Index (CHI) number, postcode, educational attainment, family history of psychosis, psychiatric admissions data, use of the Mental Health Act, current and previous medications, adverse drug effects, psychosocial interventions received and psychiatric comorbidities. All patients provide annual follow up information and there is a reciprocal relationship between local clinicians and the PsyCIS team which facilitates the return to consultants of clinically relevant information at an individual caseload level.

The dataset is regularly added to and updated and currently holds information on over 7,200 patients with a diagnosis of psychotic disorder, including: schizophrenia (n = 4,787); bipolar disorder (n = 1,784); organic psychosis (n = 67); psychotic depression (n = 452); and substance-induced psychosis (n = 160) [23]. There is a process of annual review to check the accuracy of information, such as diagnosis, postcode and current medication and the database has undergone internal validity checking to determine the diagnostic accuracy of individual ICD10 diagnoses.

### Sample

All individuals with schizophrenia or bipolar affective disorder aged between 18 and 65 who had died between 2006 and 2010 and lived within the Greater Glasgow area covered by PsyCIS were identified (n = 230). Date and cause of death were obtained by linkage to the Scottish Morbidity Records (SMR) held by the Information Services Division of NHS Scotland [24] using patient CHI number. The ICD 10 code was used to group cause of deaths, into 9 categories using information from the Scottish Government website [25]: 1) cardiovascular disease, 2) cerebrovascular disease, 3) respiratory diseases, 4) cancer, 5) alcohol related deaths, 6) mental and behavioural disorder due to drugs, 7) accidental, 8) suicide as defined by the Scottish suicide information database [26] (which includes deaths of undetermined origin) and 9) other (Table 1). Ethical Approval for this project was obtained from the NHS Greater Glasgow and Clyde Caldicott Guardian.

**Table 1:** **ICD 10 Codes for Cause of death**

**Main categories of cause of death**	**ICD 10 Codes**
1) Cardiovascular disease	I20-I25- including acute MI I219 & atherosclerotic heart disease I251 & I259
2) Cerebrovascular disease	I60-I69- including subarachnoid haemorrhage I600, I609, brain stem intra cerebral haemorrhage I613, subdural haemorrhage I620, other unspecified stroke I64 & cerebral infarction I639, I693 & I679
3) Respiratory diseases	J00-J99- including, COPD J441 & J449, pneumonia J189, J22 & J690, emphysema J439, asthma J459 & bronchiectasis J459
4) Cancer	C00-C97- including oral C069 & C329, GI tract C159, C169,19 & C20, lung C349, cervix C539, brain C719, skin C446, bladder C679 & lymphoma C819 & C851
5) Alcohol related deaths	including mental and behavioural disorder due to use of alcohol F10, degeneration of nervous system due to alcohol G31.2, alcoholic polyneuropathy G62.1, alcoholic cardiomyopathy I42.6, alcoholic gastritis K29.2, alcoholic liver disease K70, chronic hepatitis, not elsewhere classified K73, fibrosis and cirrhosis of liver K74 (excluding K74.3-K74.5 – biliary cirrhosis), alcohol induced chronic pancreatitis K86.0, accidental poisoning by, and exposure to, alcohol X45 intentional self-poisoning by, and exposure to, alcohol, X65, poisoning by, and exposure to, alcohol; undetermined intent Y15
6) Mental and behavioural disorder due to drugs	F11-F16 & F18-F19
7) Accidental	Including transport accidents V01-V99, falls W00-W19, death due to fire X00, accidental poisoning X40-X49, assault X04 & X59-Y09 and other accidents Y85 & Y86
8) Suicide	As defined by the Scottish suicide information database^17^ (X60-X84 & Y87.0 and deaths of undetermined origin Y10-Y34) including- intentional self-poisoning X61 & X62, intentional hanging X70, intentional drowning X71, intentional jump from height X80, intentional jump before moving object X81, intentional death by fire X76, poisoning of undetermined intent Y11 & Y12, fall or jump from height of undetermined intent Y30 & undetermined cause of death Y34
9) Other	Including all other codes not included in above 1-8. A00-B99, E00-E99, G00-G30, G32-G61, G62-G99, H00-H95, I00-I19, I26-I41, I43-I59, I70-I99, K00-K28, K30-KK69, K70-K85, K87-K93, L00-L99. M00-M99, N00-N99, O00-O99, Q00-Q99, R00-R99, in particular left ventricular failure I501, hypertensive disease I119, aortic aneurysm I711 & I713, peripheral vascular disease E145, ischaemic gut K550, cardiomegaly I517, cardiomyopathy I426 & I429, valvular disease I080, sarcoidosis D869, PE I269, obesity E668 & E669, unspecified cardiac arrest I469, unspecified diabetes E149, diabetic coma E140, diabetic ketoacidosis E141 and diabetes with renal complications E122, UTI N390, septicaemia A419, hypo-osmolality E871, phlebitis I803, GI haemorrhage K922, gastrointestinal ulcer K221, K254 & K264, appendicitis K37, intestinal obstruction K566 and renal failure N19

## Methods

We used the Scottish Index of Multiple Deprivation (SIMD) score as a measure of social deprivation. The SIMD identifies small areas of multiple deprivation (datazones) across Scotland by combining 38 indicators across 7 domains which are weighted. The domains include: current income, employment, health, education, geographic access to services, crime, and housing and are weighted based on evidence from Oxford University’s Social Disadvantage Research Centre [27]. Each domain contains information gathered from multiple sources, for example the health domain contains information regarding: hospital episodes related to alcohol use, hospital episodes related to drug use, a measure of mortality (the comparative mortality factor (CMF)), a measure of morbidity (comparative illness factor), emergency admission to hospital, population proportion prescribed drugs for anxiety, depression or psychosis and proportion of singleton births of low birth weight (<2,500 g). Each individual was allocated to a datazone and subsequent SIMD category based on their postcode (1 = most affluent and 5 = most deprived). There are 6,505 datazones covering Scotland and the SIMD score provides a relative measure of deprivation.

### Analyses

Rate of death/10,000 population per 5 years by SIMD quintile for the total deceased SMI population (schizophrenia and bipolar disorder combined) was calculated and compared with rate of death in the local Glasgow and wider Scottish Population over the total study time period, using mortality data available from the General Register Office for Scotland. The “Glasgow” Effect was determined by comparison of rate of death between groups. Both all-cause mortality rate and mortality rate excluding suicide in the SMI population were calculated. The rationale being that as suicide is a major contributor to premature death in SMI it may in itself be influenced by socioeconomic status. Cause of death by SIMD quintile for the total deceased SMI population (schizophrenia and bipolar disorder combined) was calculated and compared with cause of death in the local Glasgow and wider Scottish Population over the total study time period, using mortality data. Rates and patterns in cause of death by deprivation quintile were compared in those with SMI to the local Glasgow and wider Scottish populations using Chi squared tests. All statistical tests were performed using STATA version 12.

## Results

### Patterns of mortality in those with severe mental illness

The primary cause of death in those with SMI differed from that seen in the local Glasgow and wider Scottish populations (Table 2). When age at death was considered, a higher proportion of those with SMI who had died were aged 25-34 and 35-44 compared to those who had died in Scotland (7.8% vs. 5.8%, p = <0.001 and 16.5% vs. 12.5%, p = <0.001) (Table 2). The proportion of deaths by age group in those with SMI was more similar to the pattern observed locally in Glasgow (Table 2). The proportion of men that died in each group was similar: 67.8% of deaths in those with SMI were in men, 64.5% of deaths in Glasgow were in men (p = 0.298) and 61.9% of deaths in Scotland were in men (p = 0.063) (Table 2).

**Table 2:** **Rate and cause of death and socioeconomic profile of the deceased SMI group compared to Glasgow City and Scottish Populations (2006-2010)**

	**Combined SMI group (n = 230)**	**Glasgow City (n = 8,094)**	**P** ^**1**^	**Scotland (n = 54,590)**	**P** ^**2**^
**Cause of death (n, %)**					
Cerebrovascular disease	8 (3.5%)	304 (3.8%)	p = 0.833	2186 (4.0%)	p = 0.685
Cardiovascular disease	34 (14.8%	1069 (13.2%)	p = 0.546	7465 (13.7%)	p = 0.626
**Cancer**	33 (14.3%)	2211 (27.3%)	p < 0.001	18129 (33.2%)	p < 0.001
Respiratory disease	24 (10.4%)	606 (7.5%)	p = 0.096	3451 (6.3%)	p = 0.001
**Alcohol Related**	15 (6.5%)	1032 (12.8%)	p = 0.005	5319 (9.7%)	p = 0.099
Accidental deaths	11 (4.8%)	400 (4.9%)	p = 0.912	2882 (5.3%)	p = 0.734
**Suicide**	34 (14.8%)	568 (7.0%)	p < 0.001	3533 (6.5%)	p < 0.001
Mental & behavioural disorder due to drugs	18 (7.8%)	412 (5.1%)	p = 0.065	1756 (3.2%)	p < 0.001
Other	53 (23.0%)	1492 (18.4%)	p = 0.076	9869 (18.1%)	p = 0.05
**Male (n,%)**	156 (67.8%)	5218 (64.5%)	p = 0.298	33772 (61.9%)	p = 0.063
**Deaths/age group (n, %)**					
16-24	3 (1.3)	238 (2.9)	p = 0.144	1902 (3.5)	p = 0.07
**25-34**	18 (7.8)	559 (6.9)	p = 0.588	3186 (5.8)	p < 0.001
**35-44**	38 (16.5)	1250 (15.4)	p = 0.656	6841 (12.5)	p < 0.001
45-54	72 (31.3)	2171 (26.8)	p = 0.131	13841 (25.4)	p = 0.039
55-64	99 (43.0)	3876 (47.9)	p = 0.147	28820 (52.8)	p = 0.003
**Deaths/Socioeconomic Status (n, %)**					
1-Most affluent	11 (4.8%)	257 (3.2%)	p = 0.173	5974 (10.9%)	p = 0.003
2-	15 (6.5%)	450 (5.6%)	p = 0.531	8244 (15.1%)	p = 0.0003
3-	34 (14.8%)	732 (9.0%)	p = 0.003	10338 (18.9%)	p = 0.108
4-	64 (27.8%)	1286 (15.9%)	p < 0.001	12643 (23.2%)	p = 0.09
5-Most deprived	106 (46.1%)	5369 (66.3%)	p < 0.001	17391 (31.9%)	p < 0.001

P^1^ Chi squared combined SMI group vs. Glasgow City.

P^2^ Chi squared combined SMI group vs. Scotland.

Cause of death differed between the three groups: for example, death due to cancer occurred less frequently in those with SMI compared to the local Glasgow and wider Scottish populations (14.3% vs. 27.3%, p = <0.001 and 33.2%, p = <0.001) (Table 2). Alcohol related deaths were also lower (6.5% (SMI) vs. 12.8% (Glasgow), p = 0.005 and 9.7% (Scotland), p = 0.099) (Table 2). However, the proportion of suicides was higher in those with SMI compared to that seen in the local Glasgow and wider Scottish populations (14.8% vs. 7.0%, p = <0.001 and 6.5%, p = <0.001), as were drug related deaths (7.8% vs. 5.1%, p = 0.065 and 3.2%, p = <0.001) (Table 2). The proportion of deaths due to cardiovascular disease were similar across the three groups (14.8% in those with SMI, 13.2% in those living in Glasgow (p = 0.546) and 13.7% in those living in Scotland (p = 0.626).

### Patterns of mortality by socioeconomic status

**Figure 1:**
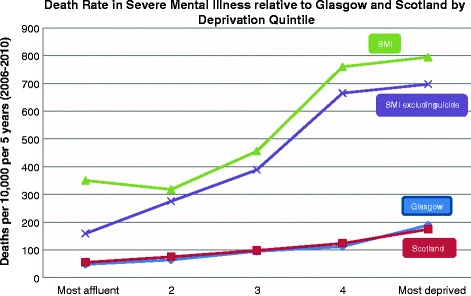
**Death Rate/10,000 population per 5 years (2006-2010).**

The rate of death per 10,000 population per 5 years for SMI compared to the local Glasgow and wider Scottish populations was higher across all socioeconomic quintiles and persisted even when suicide was excluded as a cause of death (Figure 1). For example, in the most deprived cohort, the rate of death (excluding suicide) in those with SMI was 697.2 per 10,000 population compared to a rate of 252.4 per 10,000 population for Scotland and 274.7 per 10,000 population for Glasgow. When suicide was excluded as a cause of death, the increase in mortality associated with deprivation was larger in those with SMI compared to those living in Glasgow and Scotland.

When the primary cause of death for individuals with SMI was reported in more detail by deprivation quintile, and compared to the local Glasgow and wider Scottish populations, a number of differences were identified. For those living in the most deprived cohort, higher drug-related deaths occurred in those with SMI compared to the local Glasgow and wider Scottish population rates (12.3% vs. 5.9%, p = <0.001 and 5.1% p = 0.002 respectively) (Table 3). A lower proportion of deaths due to cancer in those with SMI living in the most deprived quintile were also observed, relative to the local Glasgow and wider Scottish populations (12.3% vs. 25.1% p = 0.013 and 26.3% p = <0.001) (Table 3).

**Table 3:** **Cause of death by Socioeconomic Status: SMI compared to Glasgow and Scottish Population Bold if p<0.05**

	**Cause of Death n, (%)**	**SIMD Category**				
		**Most Affluent**				**Most deprived**
Accidental	SMI vs. Glasgow	0 (0) vs. 9 (3.5)	0 (0) vs. 21 (4.7)	1 (2.9) vs. 23 (3.1)	(7.8) vs. 59 (4.6)	5 (4.7) vs. 88 (5.4)
		p = 0.535	p = 0.40	p = 0.94	p = 0.265	p = 0.02
	SMI vs. Scotland	0 (0) vs. 273 (4.7)	0 (0) vs. 6491 (6.0)	1 (2.9) vs. 601 (5.8)	5 (7.8) vs. 618 (4.9)	5 (4.7) vs. 899 (5.2)
		p = 0.45	p = 0.345	p = 0.49	p = 0.31	p = 0.84
**Alcohol**	SMI vs. Glasgow	1 (9.1) vs. 18 (7.0)	0 (0) vs. 38 (8.4)	1 (2.9) vs. 78 (10.7)	10 (15.6) vs. 152 (11.8)	**8 (2.8) vs. 746 (13.9)**
	SMI vs. Scotland	p = 0.693	p = 0.26	p = 0.178	p = 0.425	**p = 0.003**
		1 (9.1) vs. 336 (5.8)	0 (0) vs. 530 (6.4)	1 (2.9) vs. 864 (8.4)	10 (15.6) vs. 1275 (10.1)	**8 (2.8) vs. 2314 (13.3)**
		p = 0.64	p = 0.326	p = 0.282	p = 0.196	**p = 0.004**
**Cancer**	SMI vs. Glasgow	**0 (0) vs. 109 (42.4)**	1 (6.7) vs. 163 (36.2)	1 (32.4) vs. 236 (32.2)	**8 (12.5) vs. 357 (27.8)**	**13 (12.3) vs. 1346 (25.1)**
	SMI vs. Scotland	**p = 0.03**	p = 0.067	p = 0.992	**p = 0.0312**	**p = 0.013**
		**0 (0) vs. 2726 (47.0)**	**1 (6.7) vs. 3287 (39.9)**	11 (32.4) vs. 3606 (34.9)	**8 (12.5) vs. 3942 (31.2)**	**1 (12.3) vs. 4568 (26.3)**
		**p = 0.025**	**p = 0.050**	p = 0.829	**p = 0.011**	**p = 0.008**
Cardiovascular	SMI vs. Glasgow	2 (18.2) vs. 33 (12.8)	1 (6.7) vs. 47 (10.4)	2 (5.9) vs. 109 (14.9)	8 (12.5) vs. 171 (13.1)	21 (19.8) vs. 709 (13.2)
		p = 0.44	p = 0.66	p = 0.191	p = 0.872	p = 0.09
	SMI vs. Scotland	2 (18.2) vs. 734 (12.7)	1 (6.7) vs. 1048 (12.7)	2 (5.9) vs. 1463 (14.2)	8 (12.5) vs. 1802 (19.3)	21(19.8) vs.2418 (13.9)
		p = 0.608	p = 0.525	p = 0.213	p = 0.727	p = 0.138
Cerebrovascular	SMI vs. Glasgow	1 (9.1) vs. 13 (5.1)	2 (13.3) vs. 20 (4.4)	1 (2.9) vs. 27 (3.7)	0 (0) vs. 3.4 (44)	4 (3.8) vs. 200 (3.7)
		p = 0.58	p = 0.14	p = 0.826	p = 0.139	p = 0.979
	SMI vs. Scotland	1 (9.1) vs. 253 (4.4)	2 (13.3) vs. 378 (4.6)	1 (2.9) vs. 390 (3.8)	0 (0) vs. 478 (3.8)	4 (3.8) vs. 687 (4.0)
		p = 0.455	p = 0.138	p = 0.806	p = 0.120	p = 0.929
**Mental & behavioural disorder due to drugs**	SMI vs. Glasgow	0 (0) vs. 4 (1.6)	**2 (13.3) vs. 12 (2.7)**	2 (5.9) vs. 23 (3.1)	1 (1.6) vs. 58 (4.5)	**13 (12.3) vs. 315 (5.9)**
		p = 0.68	**p = 0.028**	p = 0.40	p = 0.275	**p <0.001**
	SMI vs. Scotland	0 (0) vs. 72 (1.2)	**2 (13.3) vs. 1.7 (138)**	2 (5.9) vs. 238 (2.3)	1 (1.6) vs. 422 (3.3)	**13 (12.3) vs. 886 (5.1)**
		p = 0.716	**p = 0.001**	p = 0.183	p = 0.44	**p = 0.002**
Other	SMI vs. Glasgow	1 (9.1) vs. 4.5 (17.5)	5 (33.3) vs. 91 (20.2)	8 (23.5) vs. 133 (18.2)	16 (25.0) vs. 258 (20.1)	23 (21.2) vs. 965 (18.0)
		p = 0.52	p = 0.34	p = 0.521	p = 0.44	p = 0.418
	SMI vs. Scotland	1 (9.1) vs. 1043 (18.0)	5 (33.3) vs. 1447 (17.6)	8 (23.5) vs. 1899 (18.4)	16 (25.0) vs. 2381 (1838)	23 (21.2) vs. 3099 (17.8)
		p = 0.525	p = 0.207	p = 0.528	p = 0.310	p = 0.393
Respiratory	SMI vs. Glasgow	0 (0) vs. 11 (4.3)	2 (13.3) vs. 25 (5.6)	3 (8.8) vs. 45 (6.1)	8 (12.5) vs. 98 (7.6)	11 (10.4) vs. 427 (8.0)
		p = 0.49	p = 0.257	p = 0.287	p = 0.199	p = 0.405
	SMI vs. Scotland	0 (0) vs. 217 (3.7)	2 (13.3) vs. 406 (4.9)	3 (8.8) vs. 621 (6.0)	8 (12.5) vs. 888 (7.0)	11 (10.4) vs. 1319 (7.6)
		p = 0.527	p = 0.169	p = 0.522	p = 0.121	p = 0.322
**Suicide**	SMI vs. Glasgow	**6 (54.6) vs. 15 (5.8)**	2 (13.3) vs 33 (7.3)	5 (14.7) vs. 58 (7.9)	8 (12.5) vs. 82 (6.9)	**13 (12.3) vs. 373 (6.9)**
		**p < 0.001**	p = 0.433	p = 0.208	p = 0.08	**p = 0.054**
	SMI vs. Scotland	**6 (54.6) vs. 320 (5.5)**	2 (13.3) vs. 519 (16.3)	5 (14.7) vs. 656 (6.3)	8 (12.5) vs. 837 (6.6)	**13 (12.3) vs. 1201 (6.9)**
		**p < 0.001**	p = 0.308	p = 0.07	p = 0.086	**p = 0.05**
All Cause of death	SMI vs. Glasgow	11 vs. 257	15 vs. 450	34 vs. 732	64 vs. 1286	106 vs. 5369
	SMI vs. Scotland	11 vs. 5947	15 vs. 8244	34 vs. 10388	64 vs. 12643	106 vs. 17391

The proportion of suicide was significantly higher in those with SMI living in the more affluent quintiles relative to the local Glasgow and wider Scottish populations (54.6% vs. 5.8%, p = <0.001 and 5.5%, p = <0.001) (Table 3). Similarly, an increase in proportion of suicide was observed for those with SMI living in the most deprived quintile compared to the local Glasgow and wider Scottish population (12.3% vs. 6.9%, p = 0.05 and 6.9%, p = 0.05) (Table 3).

## Discussion

Our cohort of individuals with SMI had higher mortality rates compared to both the local Glasgow and wider Scottish populations, consistent with several other studies which have used similar secondary care mental health registers [28-30]. The proportion of deaths by age group was similar to that seen in the local Glasgow population, but differed from that of the wider Scottish population, with a larger proportion of deaths occurring in those aged 25-44 years with SMI. As occurs in the general population, increasing socioeconomic deprivation appeared to be associated with an increased overall rate of death in those with SMI.

### Differences in the rate, age and cause of death

We found that although individuals with SMI died younger than the Scottish population, the age at death was similar to the local Glasgow population. This is potentially in keeping with the “Glasgow” Effect whereby average age at death in Glasgow is lower than the Scottish average. However despite this, differences in cause and rate of death between the SMI and Glasgow groups were apparent. In particular, and perhaps surprisingly, deaths due to cancer and alcohol were lower in the SMI group compared to the Glasgow group. To date, the literature surrounding cancer deaths in individuals with SMI has been mixed, with some studies reporting elevated rates [31], while others report lower rates of cancer deaths [32]. A recent meta-analysis of incidence of cancer [33], reported that pooled overall rates of cancer incidence were not significantly increased in individuals with schizophrenia compared to controls (Standardised Incidence Rate (SIR) 1.05, 95% CI 0.95-1.15) and although incidence of lung cancer was increased, when adjusted for smoking there was no difference in incidence rates. Indeed it also reported that the incidence of several cancers unrelated to smoking was reduced in individuals with schizophrenia. These mixed findings in cancer rates may in part, help explain the unclear evidence for cancer mortality rates in individuals with SMI and highlight the need for further longitudinal research in this area.

Our finding of lower rates of death due to alcohol are perhaps surprising given the high rates of alcohol dependence reported in individuals with SMI [34,35]. This may in part be explained by the elevated population levels of alcohol related deaths in Scotland compared to other countries. In 2011 the Scottish male alcohol mortality rate was 28.4/100,000 compared to the UK average of 17.2/100,000 while in women the Scottish alcohol mortality rate was 13.9/100,000 compared to the UK average of 8.3/100,000. [36] Our finding of a trend towards significantly higher proportions of drug related deaths and “other” causes of death (which included a wide range of causes) within the SMI cohort may also explain this finding. Given that only the primary cause of death was obtained from the GRO, rates of alcohol as a secondary cause of death within our cohort are unknown and may also explain our lower rates of death due to alcohol.

We also found that the proportion of cardiovascular disease deaths in those with SMI was only marginally higher compared to Glasgow and Scotland. This is in keeping with other studies which have found similar rates of cardiovascular deaths in patients with SMI compared to their local population [29].

### The role of deprivation

We found that, as with the local Glasgow and wider Scottish populations, rate of death in those with SMI increased as deprivation increased. The magnitude of the elevation in mortality associated with deprivation was similar; however, when suicide was excluded as a cause of death, the magnitude of increase in mortality associated with deprivation was greatest for the SMI cohort. This association between deprivation and mortality in those with SMI appear similar to that reported in the general population. Of note, was our finding of a significantly higher proportion of suicide in those with SMI in both the least and most deprived quintiles. While a negative correlation between suicide and socioeconomic status has been found in Australia [37] and the US [38], our finding of a more complex relationship has been identified by others in the UK [39,40]. In particular, Osborn and colleagues found that although suicide rates were increased across all age groups of individuals with SMI, those who were least socially deprived were particularly at risk [41].

Despite the negative impact of deprivation on health outcomes (for example, those living in the most deprived areas are more likely to have unplanned admissions to hospital than those living in the most affluent areas) [42] as well as the premature onset of multimorbidity [42], studies investigating the relationship between socioeconomic status and mortality in individuals with SMI are limited. While there are studies looking at the impact of socioeconomic status on parasuicide [43], suicide [40,44] and cause of death in major depressive disorder [45,46], generalised anxiety disorder [47] and alcohol misuse [48], we found only one study which specifically focused on the impact of socioeconomic status on mortality in bipolar disorder [49] and no studies which specifically focused on the impact of socioeconomic status on mortality in schizophrenia. The study which explored the impact of socioeconomic factors on cause and rate of death in affective disorders was limited due its small sample size of only 30 deaths.

### The “Scottish” and the “Glasgow” effects

Although life expectancy and health behaviours have improved in Glasgow over the past 50 years, the rates of improvement have differed depending on the individuals’ socioeconomic status. As Scotland’s ranking in terms of European mortality rates have worsened over time, so too has the gap in life expectancy between the most affluent and most deprived areas within Scotland [50,51]. This health inequality associated with socioeconomic status has been mirrored by an inequality in life expectancy associated with SMI [10-14].

Although the impact of deprivation on health and mortality is marked, the differences in life expectancy in Scotland compared to the rest of Europe, and in Glasgow compared to the rest of Scotland, cannot be fully explained by deprivation alone. These gaps in mortality which cannot be explained by deprivation have been termed the “Scottish” and “Glasgow Effects” [52,53]. This “Glasgow Effect” was reflected in our study, where mortality rates for those with SMI were even higher than both the Scottish and Glasgow averages. The differences in mortality between those with SMI and those without also appeared largest for those living in the most deprived quintiles.

Our study of all-cause mortality in those with SMI in Glasgow has identified a trend towards increasing mortality associated with worsening deprivation, but more work is required to investigate this relationship further.

### Strengths & limitations

Our findings of a gradient in level of deprivation and magnitude of increase in mortality in those with SMI are novel in that we were able to describe the rate and cause of death of individuals with SMI in detail and compare them to both the local Glasgow and wider Scottish populations. To our knowledge, this is the first study to investigate the impact of deprivation on rate and cause of death of those with schizophrenia and bipolar disorder and as such may help inform the development of population-based strategies to improve the life expectancy of individuals with SMI. Additional strengths of this study include the comprehensive nature of the PsyCIS database, which is representative of a large clinical population accessing mental health services in a defined urban area. Regular checks of data accuracy are carried out by the senior medical practitioners involved in case management, thereby maintaining the reliability of diagnoses, clinical and sociodemographic data.

Limitations of this work include the relatively small number of deaths which occurred during our limited study timeframe. Furthermore, exclusion of a small number of patients with psychosis who are managed exclusively in primary care also occurred. As PsyCIS is a secondary care based database, inclusion of all people with psychosis in the geographical area would require linkage of the database to primary care records, which is not currently possible. Although some patients with psychosis live independently in the community without input from secondary services, further study is required to ascertain the numbers of such patients in Glasgow. PsyCIS also excludes individuals who are under 16, over 65 years old and who are not managed by general adult community services, such as those whose psychotic illness is managed exclusively in addictions psychiatry, old age psychiatry, or learning disability services. These numbers are likely to be relatively small because the majority of patients of working age with psychotic disorder are managed by adult general psychiatric services. SIMD is a relative measure of deprivation and given the impact of socioeconomic drift in the PsyCIS cohort, wider Glasgow and Scottish population during our five year timeframe, its use has a number of limitations. A further limitation was that of the impact of age on rates and causes of death- although adjustment for age profile of the populations were made, full standardisation was not possible due to small sample sizes.

## Conclusion

In our cohort of patients with SMI the rate and cause of death differed from that of the local Glasgow and wider Scottish populations. There appeared to be a graded effect of deprivation on mortality, which was largest in impact for those with SMI when suicide was excluded as a cause of death. This pattern of rising mortality across deprivation categories may represent evidence of inequalities in health outcomes for the most deprived individuals with SMI. More work is required to investigate this relationship further, in order to inform new approaches to health care organisation, health promotion and screening in those with SMI living in the most deprived communities.

## Abbreviations

GRO: General register office
ICD10: International classification of disease
PsyCIS: Glasgow psychosis clinical information system
SIMD: Scottish index of multiple deprivation
SMI: Severe mental illness
SMR: Standardised mortality rates
